# Drug-Induced Conformational Dynamics of P-Glycoprotein Underlies the Transport of Camptothecin Analogs

**DOI:** 10.3390/ijms242216058

**Published:** 2023-11-07

**Authors:** Gershon A. K. Mensah, Katherine G. Schaefer, Michael G. Bartlett, Arthur G. Roberts, Gavin M. King

**Affiliations:** 1 Department of Pharmaceutical and Biomedical Science, University of Georgia, Athens, GA 30602, USA; gershon.akesemensah@uga.edu (G.A.K.M.);; 2 Department of Physics and Astronomy, University of Missouri, Columbia, MO 65211, USA; kgsqp8@mail.missouri.edu; 3 Joint with Department of Biochemistry, University of Missouri, Columbia, MO 65211, USA

**Keywords:** membrane protein, lipid bilayer, nuclear magnetic resonance spectroscopy, force microscopy

## Abstract

P-glycoprotein (Pgp) plays a pivotal role in drug bioavailability and multi-drug resistance development. Understanding the protein’s activity and designing effective drugs require insight into the mechanisms underlying Pgp-mediated transport of xenobiotics. In this study, we investigated the drug-induced conformational changes in Pgp and adopted a conformationally-gated model to elucidate the Pgp-mediated transport of camptothecin analogs (CPTs). While Pgp displays a wide range of conformations, we simplified it into three model states: ‘open-inward’, ‘open-outward’, and ‘intermediate’. Utilizing acrylamide quenching of Pgp fluorescence as a tool to examine the protein’s tertiary structure, we observed that topotecan (TPT), SN-38, and irinotecan (IRT) induced distinct conformational shifts in the protein. TPT caused a substantial shift akin to AMPPNP, suggesting ATP-independent ‘open-outward’ conformation. IRT and SN-38 had relatively moderate effects on the conformation of Pgp. Experimental atomic force microscopy (AFM) imaging supports these findings. Further, the rate of ATPase hydrolysis was correlated with ligand-induced Pgp conformational changes. We hypothesize that the separation between the nucleotide-binding domains (NBDs) creates a conformational barrier for substrate transport. Substrates that reduce the conformational barrier, like TPT, are better transported. The affinity for ATP extracted from Pgp-mediated ATP hydrolysis kinetics curves for TPT was about 2-fold and 3-fold higher than SN-38 and IRT, respectively. On the contrary, the dissociation constants (*K*_D_) determined by fluorescence quenching for these drugs were not significantly different. Saturation transfer double difference (STDD) NMR of TPT and IRT with Pgp revealed that similar functional groups of the CPTs are accountable for Pgp-CPTs interactions. Efforts aimed at modifying these functional groups, guided by available structure-activity relationship data for CPTs and DNA-Topoisomerase-I complexes, could pave the way for the development of more potent next-generation CPTs.

## 1. Introduction

P-glycoprotein (Pgp) is a member of the ATP-binding cassette (ABC) superfamily that plays a crucial role in the development of multi-drug resistance (MDR) in chemotherapy [1]. This transporter protein is believed to be present in more than 50% of tumor cells in patients undergoing chemotherapy [2]. Studies have demonstrated a direct correlation between Pgp expression levels in cancer patients and adverse clinical outcomes in chemotherapy [3]. Evolutionarily, Pgp plays a vital role in safeguarding cells against toxic insults [4,5]. Thus, apical plasma membranes of cells that constitute essential physiological barriers and excretory tissues, including the blood-brain barrier, kidney, and liver, exhibit high expression levels of Pgp [6,7]. In a self-adaptive defense mechanism, tumor cells tend to increase the expression of Pgp to evade cell death [8,9,10]. Regrettably, most chemotherapeutic agents are substrates of Pgp, a phenomenon that contributes significantly to the development of MDR phenotypic traits observed in cancer patients [11,12]. By actively pumping life-saving chemotherapeutic drugs out of cells, Pgp decreases the intracellular concentrations of drugs below the therapeutic levels required to be effective. Despite significant advancements in technology and research, the resistance of human malignancies to multiple chemotherapeutic agents remains a considerable barrier in cancer therapy [2,13] and potentially accounts for over 80% of treatment failures in patients on chemotherapy [14].

Camptothecin (CPT) is a molecular scaffold for a class of anti-cancer drugs that effect its toxicity by targeting topoisomerase I [15,16]. During the S phase of the cell cycle, camptothecin and its semi-synthetic derivatives (CPTs) selectively attach to and stabilize the topoisomerase-I-DNA complexes at the replication fork, resulting in potentially fatal breaks in DNA. [15,17]. In addition, new evidence suggests that CPTs may induce antitumor effects by alternative mechanisms, such as ubiquitin/26S proteasome-mediated degradation of topoisomerase-1 proteins [18]. In general, the CPTs exist in two different pH-driven forms in solution—lactone and carboxylate [19]. The ability to passively diffuse into cells and exhibit antitumor properties is attributed to the lactone form [20,21].

Topotecan (TPT) and Irinotecan (IRT) are FDA-approved semi-synthetic CPTs frequently utilized as first- and second-line treatments for various types of cancer, including colorectal carcinoma [15]. TPT and IRT are more soluble, have improved bioavailability, and are less toxic compared to the parent compound [22,23]. IRT is a prodrug activated by the enzyme carboxylesterase to produce its active metabolite, SN-38, which is 100 to 1000 times more potent than the prodrug [23]. Given the crucial role of CPTs as effective anti-cancer drugs in chemotherapy, it is unsurprising that several other analogs, such as 9-amino camptothecin, are currently being developed and tested in various clinical trials [15]. Unfortunately, these analogs may suffer the same fate as the currently available antitumor agents if Pgp significantly transports them.

**Figure 1:**
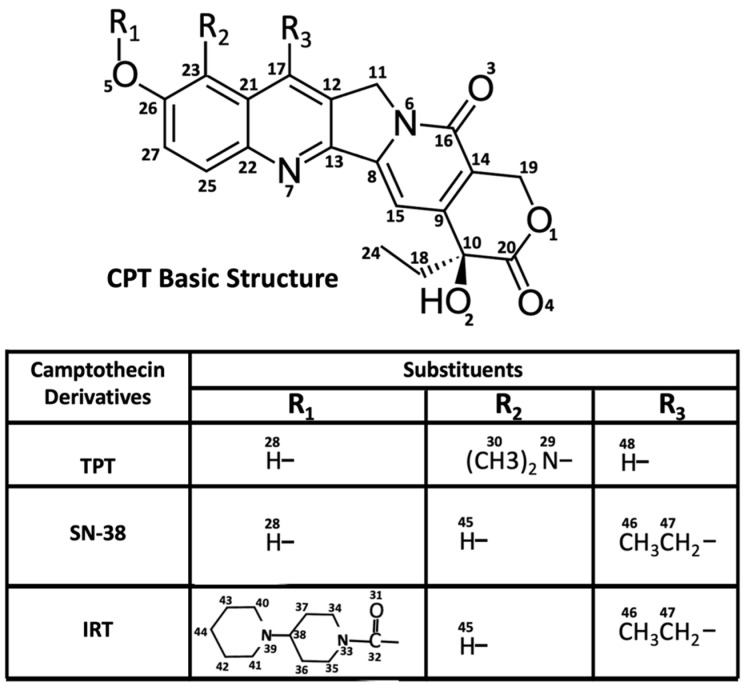
The basic structure of the camptothecin analogs. The table shows distinctions between TPT, SN-38, and its pro-drug, IRT.

Although TPT, IRT, and SN-38 share the same basic structure, as illustrated in Figure 1, their pharmaceutical and pharmacological characteristics differ significantly [15]. Several membrane transporters can restrict the availability and distribution of drugs, including the CPTs [24]. Pgp is a predominant efflux pump that eliminates CPTs from cancer cells [25,26]. A retrospective clinical study on second-line chemotherapy revealed that patients treated with IRT had a higher survival rate than those treated with TPT [27]. Previous ex vivo studies have shown that IRT demonstrated greater efficacy than TPT in different human tumor xenograft models [28]. In addition, SN-38 has been reported to have >2-fold higher cellular retention than TPT [29], while IRT showed a 2-fold higher tissue accumulation rate than SN-38 [30]. In vitro analysis using Pgp-transfected Caco-2 cells suggests that TPT has a Pgp-mediated efflux ratio about 6-fold higher than IRT [31]. In LS513 cells treated with BCRP and MRP2 inhibitors, the relative efflux rate of TPT was approximately 4 times higher than that of SN-38 [32]. These results, taken together, suggest that the order of relative Pgp-mediated efflux rates for these CPTs is TPT > SN-38 > IRT. However, the different cell lines and methods used in these transport measurements make detailed analysis and comparison difficult. Another significant drawback in these studies was the authors’ inability to account for the mechanisms underlying the observed differences in reported efflux rates. So little is known about the CPTs that even the functional groups that interact with Pgp are yet to be identified.

Our primary objective in this study was to identify the functional groups of CPTs that interact with Pgp. Identifying and modifying the functional groups could lead to the development of next-generation CPT analogs that have lower affinity for Pgp and are, therefore, better able to evade the transporter. Such analogs may have improved cellular retention and potency. Further, we investigated the molecular and structural mechanisms that drive Pgp-mediated transport of CPTs. Understanding the underlying mechanisms driving Pgp-mediated efflux is crucial for facilitating rational drug design aimed at circumventing Pgp-mediated MDR. To perform this, we explored a potential drug-ATP cooperativity by measuring Pgp-mediated ATP hydrolysis in the presence of varying concentrations of TPT, IRT, and SN-38. To investigate the structural mechanisms underlying Pgp-mediated efflux of TPT, IRT, and SN-38, we examined the drug-induced conformational changes in Pgp. These conformational changes were studied using fluorescence spectroscopy and Atomic Force Microscopy (AFM). The binding affinity of CPTs to Pgp was estimated by probing the quenching of intrinsic Pgp fluorescence by the drugs. Finally, NMR saturation transfer double difference (STDD) was employed to identify functional groups on these drugs crucial in Pgp-drug interactions.

## 2. Results

### 2.1. Effect of Topotecan, Irinotecan, and SN-38 on Pgp-Coupled ATPase Activity

Pgp-mediated efflux is an ATP-dependent process that releases inorganic phosphates from ATP hydrolyses [33]. Previous studies suggest that Pgp requires the hydrolysis of at least one molecule of ATP per complete catalytic cycle [34,35]. Substrate-stimulated ATPase activity assays have been used previously to identify Pgp substrates/inhibitors [36,37] and predict the rate of effective transport of substrates [38]. For most substrates, the rate of Pgp-coupled ATP hydrolysis correlates with transport [39,40]. An ATPase activity assay was used to determine the coupling effect of the rate of ATP hydrolysis on the Pgp-mediated transport of CPTs.

**Figure 2:**
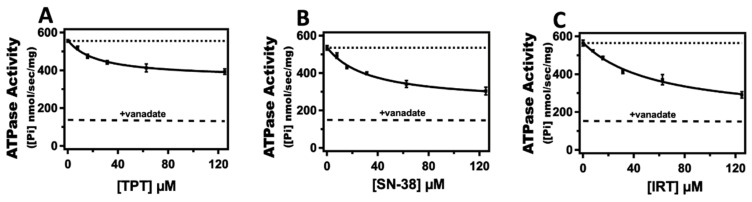
The effect of TPT, SN-38, and IRT on Pgp-mediated ATP hydrolysis. Ligand-induced Pgp-coupled ATPase activity in the presence of varying concentrations of (**A**) TPT (closed squares), (**B**) SN-38 (closed squares), and (**C**) IRT (closed squares). For comparison, each panel shows the basal activity level recorded in the absence of ligands as a dotted line. The dashed line in each panel represents the average ATPase activity in the presence of 200 µM of vanadate. Each data point represents the average of at least three independent experiments, and the error bars shown represent the standard deviation.

Figure 2 shows the effect of TPT, SN-38, and IRT on Pgp-mediated ATP hydrolysis. The kinetic curves generated from Pgp-mediated ATPase hydrolysis in the presence of TPT, IRT, and SN-38 were monophasic, suggesting a single drug binding site for these CPTs on the protein. In the absence of drugs (basal conditions), the average rates of Pgp-coupled ATP hydrolysis were similar, averaging 555 ± 9 nmol min^−1^ mg^−1^ (Figure 2). These basal rates of ATPase activities observed align well with the range observed in previous studies [41,42,43]. Kinetic parameters were determined by fitting Pgp-coupled ATP hydrolysis kinetic curves into a modified Michaelis-Menten equation (Equation (1)) that accounts for the basal activity. For TPT (Figure 2A), the estimated *K_m_* from the kinetic curve was 21.51 ± 4.56 µM, closely resembling a previously reported value observed in CaCo-2 cells [44]. The *V_max_*, representing the maximum rate of ATPase activity at a saturating drug concentration, was estimated at 360 ± 12 nmol min^−1^ mg^−1^ for TPT. As for SN-38 (Figure 2B), the calculated *K_m_* and *V_max_* were 42.90 ± 7.93 µM and 242 ± 10 nmol min^−1^ mg^−1^, respectively. In the case of IRT (Figure 2C), the *K_m_* and *V_max_* were 58.52 ± 14.81 µM and 167 ± 12 nmol min^−1^ mg^−1^, respectively. Although most Pgp substrates stimulate Pgp-mediated ATPase activity, a few have been reported to reduce it below basal levels [39,45,46]. The ability to influence the rate of Pgp-mediated ATP hydrolysis in a dose-dependent, saturable manner, as seen with TPT, SN-38, and IRT, is a key characteristic shared by most Pgp substrates [44,47]. These findings indicate that while CPTs reduce the ATPase activity rate below the basal level, the extent of reduction differs for each drug within the class. Overall, these results imply that the mechanisms underlying Pgp-mediated transport of CPTs also influence the rate of drug-induced Pgp-mediated ATP hydrolysis.

### 2.2. Determination of Drug-Pgp Binding Affinity by Protein Intrinsic Tryptophan Fluorescence Quenching

**Figure 3:**
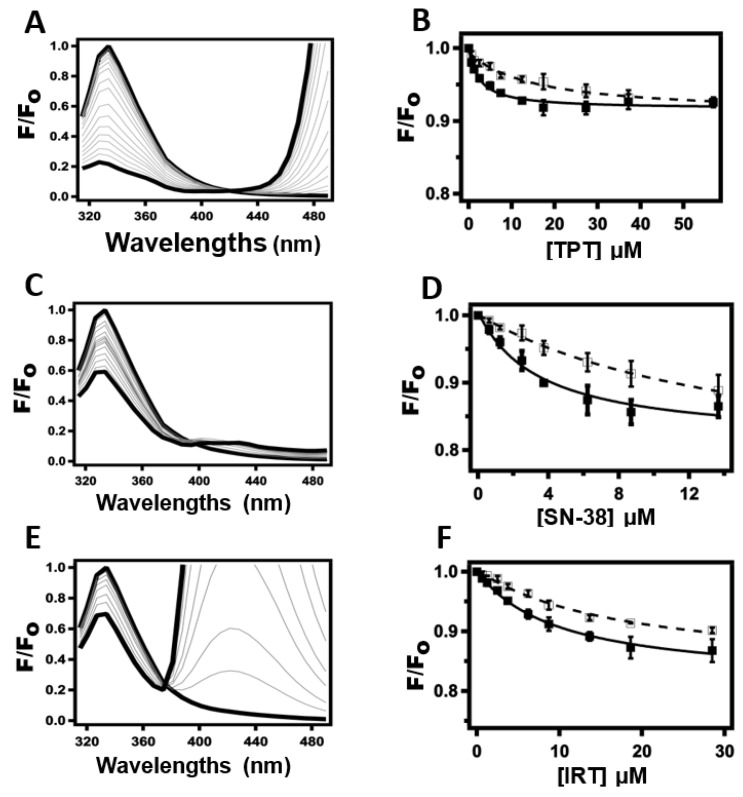
The binding affinity of TPT, SN-38, and IRT to Pgp was determined by the quenching of intrinsic protein fluorescence. Fluorescence emission decay spectra of Pgp following excitation at 295 nm in the presence of an increasing concentration of (**A**) TPT, (**C**) SN-38, and (**E**) IRT. The upper and lower thick lines represent the absence (0 µM) and the presence of saturating TPT concentrations, respectively. Fluorescence spectra at intermediate concentrations are shown in gray lines. The corrected fluorescence emission amplitudes at 330 nm were plotted as a function of the concentration of (**B**) TPT, (**D**) SN-38, and (**F**) IRT in the absence (closed squares) and presence (open squares) of 3.2 mM AMPPNP. Each data point and error bar represent the average and standard deviation of at least three independent experiments.

The measurement of intrinsic protein fluorescence quenching by ligands is a robust spectrophotometric tool that has been used to determine protein-ligand binding affinities in previous studies [48,49]. This technique was used to determine the apparent binding affinity of TPT, IRT, and SN-38 to Pgp. Figure 3 shows the analysis of Pgp fluorescence in the presence of increasing concentrations of TPT, SN-38, and IRT. Panels 3A, 3C, and 3E show the normalized Pgp fluorescence emission spectrum deduced in the presence of increasing concentrations of TPT, SN-38, and IRT, respectively. The protein fluorescence spectra showed a maximum fluorescence at 330 nm following excitation at 295 nm, whereas the tested CPTs showed significant fluorescence beyond 380 nm in a dose-dependent manner. In panels 3A, 3C, and 3E, the thick upper lines indicate the highest protein fluorescence levels observed in the absence of drugs. In the presence of 100 µM of TPT (Figure 3A, lower thick lines), 60 µM of SN-38 (Figure 3C, lower thick lines), and 100 µM IRT (Figure 3E, lower thick lines), protein fluorescence decreased by approximately 71.6%, 40.6%, and 21.0%, respectively, from the initial fluorescence. The CPTs tested in this study were relatively insoluble in aqueous solutions. The lower, thicker lines show the minimum protein fluorescence possible before any visible precipitation. In the presence of saturating AMPPNP, 100 µM TPT, 60 µM SN-38, and 100 µM IRT decreased the protein fluorescence by 61.0%, 38.8%, and 21.7%, respectively. Figure 3B,D,F show Pgp fluorescence monitored at 330 nm following excitation at 295 nm for increasing concentrations of TPT, SN-38, and IRT, respectively. Pgp fluorescence amplitudes at 330 were corrected for the inner filter effect using Equation (2). The corrected fluorescence amplitudes (*F*_(*corrected*)_) were plotted against the concentration of TPT (Figure 3B), SN-38 (Figure 3D), and IRT (Figure 3F) in the absence (closed squared) and presence (opened squares) of saturating (3.2 mM) AMPPNP. The protein fluorescence in the presence of the CPTs analyzed in this study, either in the presence or absence of AMPPNP, showed a monophasic curve, which implies a single binding site on the Pgp and is consistent with our other results. Subsequently, the protein fluorescence kinetic curves generated were fitted to Equation (3) to estimate the Stern–Volmer’s constant (*K_sv_*). The maximum concentrations of the CPTs used for fitting were the highest permissible, given the notable inner filter effect exhibited by these drugs. The *K_sv_* values from the CPTs-induced Pgp fluorescence curve for TPT, SN-38, and IRT in the absence of AMPPNP were 0.41 ± 0.09 µM^−1^, 0.28 ± 0.09 µM^−1^, and 0.18 ± 0.01 µM^−1^, respectively. In the presence of saturating AMPPNP, the estimated *K_sv_* values decreased to 0.06 ± 0.01 µM^−1^, 0.076 ± 0.02 µM^−1^, and 0.07 ± 0.01 µM^−1^ for TPT, SN-38, and IRT, respectively. Since the *K_sv_* values for the CPTs evaluated decreased significantly at a higher temperature (37 °C), static rather than dynamic quenching was projected to be the dominant mechanism underlying the protein fluorescence quenching observed. For static quenching, the *K_sv_* value correlates with the ligand-protein binding affinity [50,51]. The estimated *K*_D_ for TPT (Figure 3B) in the absence and presence of saturating AMPPNP were 2.47 ± 0.57 µM^−1^ and 16.03 ± 3.27 µM^−1^, respectively. The *K*_D_ recorded for SN-38 (Figure 3D) was 3.53 ± 1.14 µM^−1^, which increased to 13.20 ± 4.23 µM^−1^ in the presence of saturating AMPPNP. For IRT (Figure 3F), the *K*_D_ was determined to be 5.71 ± 1.65 µM^−1^, which increased to 14.92 ± 2.39 µM^−1^ in the presence of AMPPNP.

### 2.3. Probing TPT, SN-38, and IRT-Induced Pgp Conformational Changes Using Acrylamide Quenching of Protein Fluorescence

The tertiary structure of a protein may undergo conformational changes due to its interactions with ligands [51,52,53]. Pgp-mediated translocation of substrates across the lipid bilayer has been reported to be modulated by ligand-induced conformational changes in the protein [54]. Acrylamide quenching of protein fluorescence is a spectrophotometric technique that has been used in previous studies to indirectly determine ligand-induced changes in the tertiary orientations of proteins [55,56,57]. The degree of acrylamide quenching is hypothesized to be highest when the protein is in the open-inward conformation [58,59] and least when the protein is in the open-outward conformation [43,58,59]. Acrylamide quenching of protein fluorescence was used to test the hypothesis that Pgp-mediated translocation of TPT, SN-38, and IRT across the lipid bilayer is modulated by ligand-induced conformational changes in the protein.

**Figure 4:**
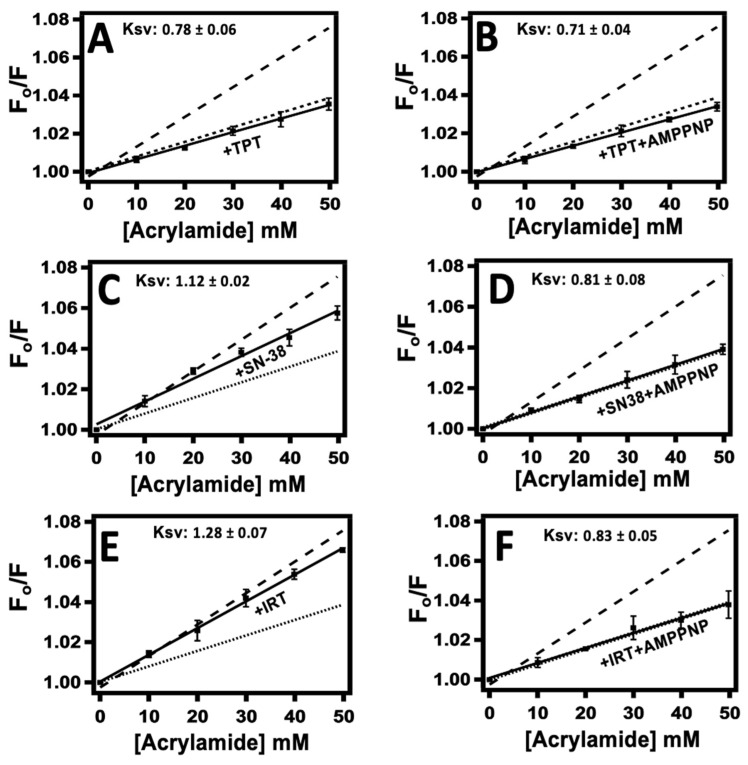
Camptothecin-induced conformational changes in Pgp are probed by acrylamide quenching of protein fluorescence. Stern–Volmer plots of Pgp induced by TPT are shown in the (**A**) absence and (**B**) presence of saturating AMPPNP. (**C**) and (**D**) show SN-38-induced Stern–Volmer plots of Pgp in the presence and absence of AMPPNP, respectively. The Stern–Volmer plots of Pgp induced by IRT are shown in (**E**) absence and (**F**) presence of AMPPNP. For reference, the Stern–Volmer plot of apoPgp in the absence (dashed line) and the presence of AMPPNP (dotted line) are shown in each panel. The TPT, SN-38, IRT, and AMPPNP concentrations used were 60 µM, 30 µM, 100 µM, and 3.2 mM, respectively. Shown in each panel is the Ksv value deduced by fitting the Stern–Volmer plot to Equation (3). The data points and error bars represent the mean and standard deviation for at least three independent experiments.

Figure 4 shows the Stern–Volmer plots of drug-induced quenching of Pgp fluorescence by acrylamide in the presence of 60 µM TPT, 30 µM SN-38, and 100 µM IRT. The *K_sv_* values were determined by fitting the Stern–Volmer kinetic curve into Equation (4) after accounting for the inner filter effect, dilution, and background with Equation (2). This value represents the slope and provides an estimate of the extent of quenching. Given that acrylamide is a small polar molecule with limited ability to penetrate the protein’s hydrophobic core, a higher *K_sv_* value indicates increased exposure of the tryptophan residues to the surrounding solvent. Acrylamide was titrated against the protein in the presence of the tested CPTs and saturating (3.2 mM) AMPPNP to assess the potential cooperative effects of AMPPNP on CPT-induced conformational changes in Pgp. The *K_sv_* value obtained from the Stern–Volmer plot of NATA (S1A, dash-dotted line) represents a positive control and provides insight into the highest possible degree of quenching had all the tryptophan residues been fully exposed to the solvent. The estimated *K_sv_* value for NATA was 15.43 ± 0.37 M^−1^, which correlates with prior studies [60,61,62]. The estimated *K_sv_* values for apoPgp (S1B, dash-line) and Pgp in the presence of AMPPNP (S1B, dotted-line) were 1.58 ± 0.03 M^−1^ and 0.84 ± 0.07 M^−1^, respectively, consistent with previously published data [63,64]. Note that the significantly lower *K_sv_* value observed for apoPgp compared to NATA indicates that only a fraction of the tryptophan residues within Pgp are accessible by the bulk solvent. The X-ray structures of mammalian Pgp and similar bacterial transporters devoid of ligands depict the transporter in a predominantly open-inward state, with notable distance between the two nucleotide-binding domains (NBDs) [65,66,67]. We can infer from these findings that in the absence of ligands, Pgp predominantly adopts an open-inward conformation; thus, the *K_sv_* value deduced for apoPgp represents the protein in an open-inward conformation. However, in the presence of saturating amounts of AMPPNP, there is a noticeable shift towards a primarily open-outward orientation. Since the analysis of both the x-ray structures [68,69] and the cryo-electron microscopy structure [70] of Pgp in the presence of AMPPNP consistently revealed a conformation characterized as open-outward, it is reasonable to infer that ApoPgp adopts an open-inward conformation.

The Stern–Volmer of apoPgp (Figure 4, dashed-line) and Pgp in the presence of saturating AMPPNP (Figure 4, dotted-line) are shown in each panel for comparison. Figure 4A (solid line) and Figure 4B (solid line) show the Stern–Volmer plot of Pgp fluorescence induced by TPT in the absence and presence of AMPPNP, respectively. Figure 4C (solid line) and Figure 4D (solid line) show the effect of SN-38 on the Stern–Volmer plot of Pgp fluorescence in the absence and the presence of AMPPNP, respectively. The IRT-induced Stern–Volmer plot of Pgp was deduced in the absence (Figure 4E, solid line) and presence of AMPPNP (Figure 4F, solid line). In the absence of AMPPNP, the *K_sv_* values for TPT, SN-38, and IRT were 0.78 ± 0.06 M^−1^, 1.12 ± 0.02 M^−1^, and 1.28 ± 0.07 M^−1^, respectively. The estimated *K_sv_* values for TPT, SN38, and IRT in the presence of AMPPNP were 0.71 ± 0.04 M^−1^, 0.81 ± 0.08 M^−1^, and 0.83 ± 0.05 M^−1^, respectively. Unlike SN-38 and IRT, the introduction of a saturating concentration (3.2 mM) of AMPPNP did not have a notable effect on the *K_sv_* value induced by TPT.

### 2.4. Ligand-Receptor Interactions Determined by STDD NMR

**Figure 5:**
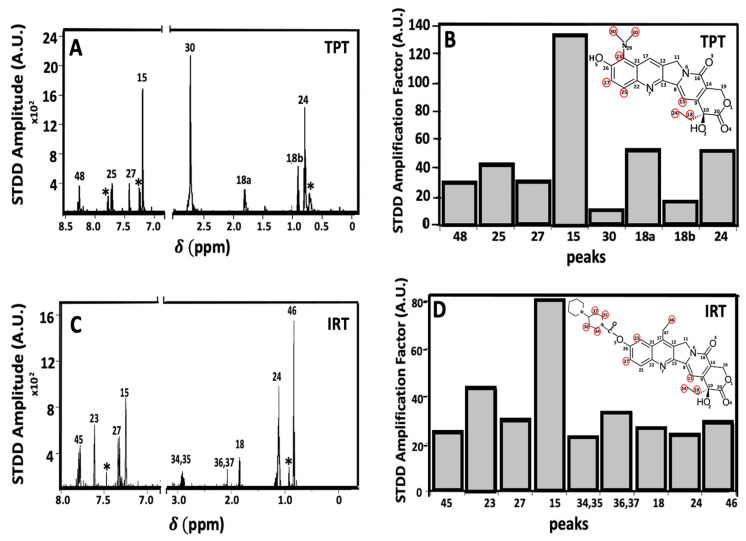
The interactions of camptothecin analogs with Pgp were investigated using STDD NMR spectroscopy. The 1D H1 STDD NMR spectra for TPT and IRT are shown in (**A**) and (**C**), respectively. Amplification factors calculated from the STDD data are shown for (**B**) TPT and (**D**) IRT. The labels on the peaks in each panel correspond to the proton labels in Figure 1. For clarity, the structures of TPT and IRT are shown in (**B**) and (**D**), respectively, and the functional groups that showed significant STDD are highlighted in red circles. The sub-labels ‘a’ and ‘b’ indicate axial and equatorial protons, respectively. The ‘*’ denotes unidentified peaks.

STDD NMR spectroscopy is a powerful technique frequently employed to determine epitope mapping and characterize ligand-receptor interactions [71,72,73]. In the present study, STDD NMR was used to identify the precise molecular moieties on TPT and IRT that likely interact directly with Pgp. STDD NMR experiments for SN-38 were not feasible due to the pronounced insolubility of most NMR-compatible solvents and instrumental limitations. Figure 5 shows the STDD NMR spectra and their corresponding amplification factors for TPT and IRT. For clarity, a labeled core structure of CPTs, accompanied by a related legend highlighting the structural similarities shared by TPT, SN-38, and IRT, is shown in Figure 1. Panels 5A and 5B show the 1D and ^1^H STDD spectra of TPT and IRT, respectively. STDD spectra for TPT and IRT were compared with their respective 1D and ^1^H spectra to identify the protons that showed significant STDD. The 1D and ^1^H spectra observed for TPT and IRT were consistent with previously reported studies [74,75,76]. In the case of TPT, the saturation transfer was most pronounced for protons from the following carbons: C_15_, C_18_, C_24_, C_25_, C_27_, C_30_, and C_48_ (see Figure 1). Similarly, concerning the interaction between IRT and Pgp, substantial saturation transfer was observed for protons associated with C_15_, C_18_, C_23_, C_24_, C_27_, C_34_, C_37_, C_45_, and C_46_ (Figure 1). These findings imply that comparable functional groups on TPT and IRT likely play a pivotal role in their interactions with Pgp. The amplification factors for TPT and IRT were determined by fitting the ^1^H-1D STDD spectral data to Equation (5). Figure 5C,D show the STDD amplification factors for TPT and IRT, respectively. The highest amplification factors for TPT and IRT were approximately 133 and 81, respectively, while their average amplification factors were around 45 and 29, respectively. IRT had a higher number of protons with notable STDD signals compared to TPT, possibly attributable to the additional cyclic hydrophobic groups inherent in IRT. These hydrophobic groups may interact weakly with the relatively hydrophobic binding pocket of Pgp [65]. In principle, saturation transfer is influenced by binding kinetics (*K*_on_ and *K*_off_), which dictate how strongly a ligand binds to a receptor and the residence lifetime of the complex [71]. A stronger ligand-receptor affinity corresponds to shorter nuclear distances, as long as the nuclear distance remains below a certain threshold. The intensity of the STDD signal has been determined to be inversely proportional to the sixth power of the distance between the nuclei [73]. Thus, a significant STDD signal intensity suggests shorter distances and tighter binding between the protein and ligand. TPT-Pgp interactions showed the highest STDD signal amplitudes, despite having a relatively smaller number of hydrophobic residues than IRT. Previous studies have shown that the amine functional groups can serve as potential hydrogen bond acceptors or donors, forming limited hydrogen bonds within the hydrophobic binding pocket of Pgp [37]. This observation may explain the relatively high STDD observed for the protons linked to C_30_ (Figure 1 and Figure 5) and the overall higher average amplification factor recorded for TPT. Overall, these results also support our Pgp-ligand affinity studies (Figure 3) that showed that, among the three CPTs under study, TPT has a relatively higher binding affinity to Pgp.

### 2.5. AFM Analysis of the Impact of Topotecan, SN-38, and Irinotecan on the Conformational Distribution of Pgp

AFM is a high-resolution imaging technique used to study the topology of many structures, ranging from single molecules to living cells [77]. This sensitive yet powerful multifunctional imaging platform has recently been used to study the conformations of proteins in the lipid layer [78,79]. Consequently, AFM imaging was adopted in this study to explore the conformational changes Pgp undergoes as it extrudes ligands (TPT, SN-38, and IRT) across the lipid bilayer. AFM imaging was used to determine the drug-induced conformational shift in Pgp and ascertain the role these changes play in the transport of the CPTs. One limitation of X-ray crystallography is its reliance on the average coordinates of atoms to determine the crystal structures of biological molecules [80]. Unlike X-ray crystal structures, AFM imaging can sample through the various conformations of a biological molecule to identify changes in the external protrusion of proteins within a membrane [81].

**Figure 6:**
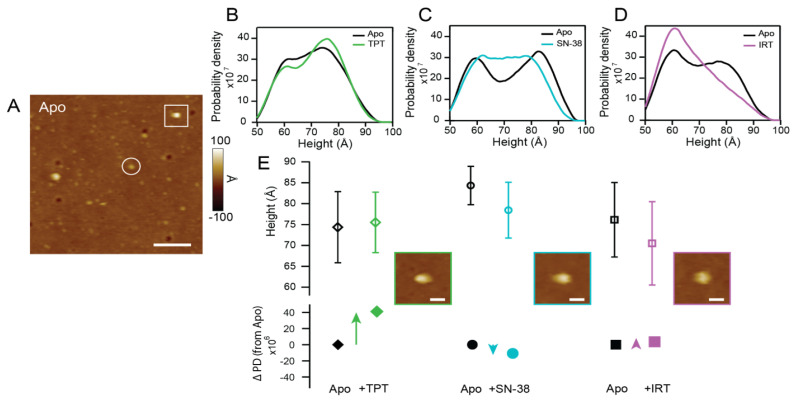
The effect of TPT, SN-38, and IRT on the height distributions of Pgp for the C-domain protrusions was determined via AFM. (**A**) An AFM image of apo Pgp indicates representative C-side (boxed) and EC-side (circled) features; scale bar = 100 nm. Smooth histograms show population shifts from apo (black) in the presence of TPT ((**B**); green), SN-38 ((**C**); blue), and IRT ((**D**); purple). Histograms are integrated into one area using SI units such that the y-axis is the probability density. (**E**) The heights of the tallest populations are compared side-by-side to show relative shifts (top row; open shapes); error bars are the standard deviation of the individual Gaussian distribution for that population. Insets show representative C-side features within the tallest population after the addition of each CPT; scale bars = 20 nm. The probability density difference (∆PD) relative to the apo state indicates how the fraction of features within these tall populations shifts in the presence of each CPT (bottom row; closed shapes).

Figure 6 illustrates the conformational shifts in Pgp induced by CPTs. A representative AFM image of Pgp in the absence of a drug that highlights both the extracellular (EC; circled) and cytosolic (C; boxed) domains is shown in Figure 6A. Following previous work, the identification of the C-domains and EC-domains of the protein in the lipid bilayer was achieved using protein height. C-side features were identified as those having heights between 55 and 90 Å. This approach has been verified using C-side-specific antibodies for Pgp (Sigdel et al., 2018). Smooth height histograms demonstrate height shifts in C-side features of Pgp in the absence and presence of TPT (Figure 6B), SN-38 (Figure 6C), and IRT (Figure 6D). The population of the EC-domain protrusions of Pgp far outnumbered the C-domain, consistent with previously reported data [79]. Conformational changes were more pronounced on the C-side. Hence, EC-side protrusions were excluded from further analysis.

After the imaging of Pgp in the absence of ligands, one of the test compounds is added to the sample and imaged so that changes in the conformation of Pgp may be observed in a nominally similar distribution of particles. This approach helps limit the inherent differences observed in samples from batch to batch. The AFM imaging data collected and analyzed fit a model selection criterion (See Supporting Information, S2). Shifts in the tallest population are closely examined because the protein’s height in the cytosolic region generally increases as the distance between the NBDs decreases, a key characteristic of Pgp in open-outward orientation. The changes in the height of Pgp at the C-side were tracked to identify the number of features occupying this state. Prior to the addition of TPT (Figure 6B), the apo state (*N* = 889) had a population centered around 74 ± 9 Å. In the presence of 60 µM TPT (*N* = 780), there was a change in height to 75 ± 7 Å, which is within error of the apo condition (Figure 6E). However, there is a significant increase in the number of features sampled at the tallest heights. We indicate this using the change in the probability density (∆PD; Figure 6E) from apo to +TPT. Generally, the height of the protein in the cytosolic region increases as the distance between the NBDs decreases, a key characteristic of Pgp in an open-outward orientation. The increase in this population, with a ∆PD of 4.11 × 10^7^, implies that TPT shifts a larger fraction of C-side features toward an open-outward conformation as compared to the apo condition. This process was repeated for the analysis of Pgp in the presence of SN-38 (Figure 6C). Apo Pgp (*N* = 217) had a Pgp with a tall C-side height population centered around 84 ± 5 Å. The addition of 30 µM SN-38 (*N* = 229) induced a decrease in the height of the taller Pgp features to 78 ± 7 Å. The fraction of features in this population decreased, with a ∆PD of −1.08 × 10^7^. Notably, the histograms show a distribution going from bimodal in the apo state to a broadened distribution in the presence of SN-38. The shift toward a more uniform histogram is, perhaps, indicative of a shift toward the intermediate conformation. Unlike the previous two drugs, IRT (Figure 6D) shifts the height distribution of Pgp to the left of the height distribution histogram, where Pgp would be in a widely open-inward conformation. The tallest population with an average C-side height of 76 ± 9 Å in the apo state (*N* = 237) decreased to 70 ± 10 Å in the presence of 100 µM IRT (*N* = 320). The ∆PD between these two populations is relatively low (4.91 × 10^6^) when compared with similar conditions involving either TPT or SN-38, suggesting that the fraction of features that exist in this population, when compared to the apo condition, is more similar.

## 3. Discussion

Pgp plays a crucial role in regulating the bioavailability of drugs and other xenobiotics, often leading to the development of MDR. To fully understand the activity of Pgp and encourage rational drug design aimed at circumventing the activity of the protein, several models that attempt to provide insight into the Pgp-mediated transport of xenobiotics have been proposed [82,83]. Some of the widely used transport models include the partitioning-alternating model [84,85,86] and ligand-ligand cooperativity [87]. A conformationally-gated model was used in this study to explain the underlying mechanism driving Pgp-mediated transport of the CPTs. Mammalian Pgp and analogous transporters in other organisms can adopt a diverse array of conformational states, both in the presence or absence of ligands [88,89]. Thus, in a lipid environment conducive to the functionality of Pgp, a broad spectrum of diverse conformations would be present. However, for clarity and simplicity, one can model Pgp into three principal conformations: ‘open-inward’, ‘intermediate’, and ‘open-outward’. The transporter protein is believed to be in an open-inward conformation when the nucleotide-binding domains are relatively separated and the drug-binding pockets are exposed to the cytosolic region. An open-outward conformation of the protein is inferred when the nucleotide-binding domains are positioned closely together and the ligand-binding cavities are accessible to the extracellular region of the cell. An intermediate conformation is established when the conformation of Pgp is between the open-inward and open-outward states, with the drug-binding region partially exposed to both the cytosolic and extracellular regions of the cell. 

Acrylamide quenching of solvent-accessible tryptophan residues in Pgp reveals that TPT caused about a 50% decrease in the *K_sv_* value of apoPgp (Figure 4), akin to the conformational change induced by AMPPNP. Since Pgp in the presence of AMPPNP [53,56] is shown to be in a predominantly open-outward conformation, we propose that TPT induces a similar conformational shift in the protein. These findings are supported by the AFM data, which revealed an increase in the population of Pgp with the tallest heights (>73 Å) when TPT was added (Figure 6B). Logically, a reduction within the population with the shortest height (<70 Å) was also observed (Figure 6B). Of the three drugs tested, TPT showed the highest probability density difference (∆PD) in the tallest populations (Figure 6E, row 2), suggesting that the C-side features adopted a more significant number of taller conformations. This finding implies that TPT induces open-outward conformation in Pgp. SN-38 and IRT caused ~28% and ~20% decreases in the *K_sv_* value of the protein, respectively, suggesting that the active metabolite of IRT does not exert as much influence on Pgp’s conformation as TPT. Analyses of the AFM height distribution histogram reveal that whereas SN-38 slightly decreased both the tallest and shortest population of Pgp (Figure 6C), IRT showed a relatively larger shift toward the left of the histogram (Figure 6D). Further, SN-38 shifted the height distribution to a nearly unimodal histogram, inferring an intermediate conformational state. In contrast, IRT and its active metabolite induced minimal overall change in the ∆PD of the tallest population (Figure 6E, row 2).

**Figure 7:**
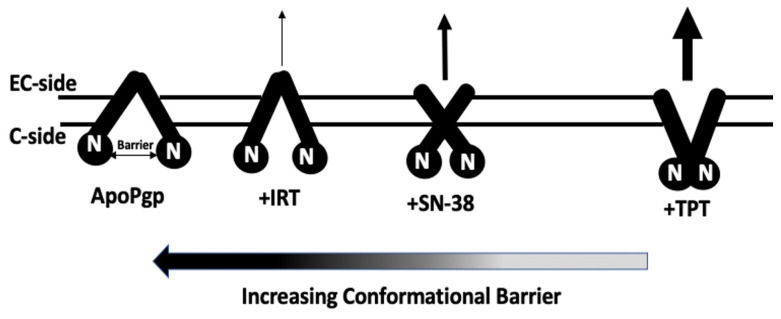
A cartoon depiction of a conformationally-gated model of Pgp conformations in the lipid bilayer in the presence of TPT, SN-38, and IRT. Pgp is represented in solid black, with the black circular region labeled ‘N’ representing the nucleotide-binding domain (NBD). The horizontal double-pointed arrow represents the distance between the NBDs and indicates the conformational barrier. The horizontal arrow shows the direction of increasing distance and conformational barrier between the two NBDs. The two horizontal lines represent the lipid bilayer. The black vertical arrows and thickness reflect the direction and rate of transport, respectively. Both the extracellular (EC) and cytosolic (C) regions are shown.

A conformationally-gated model illustrating ligand-induced changes that drive Pgp-mediated transport of CPTs based on our findings is shown in Figure 7. Drawing from our observation and previously published studies [79,90,91], we postulate that TPT induces a Pgp conformational transition from a primarily open-inward conformation to an open-outward orientation. Conversely, SN-38 shifts the protein toward an intermediate conformation, between an open-inward and an open-outward conformation. IRT, on the other hand, moderately shifts Pgp to a conformational state closer to the open-inward orientation. These transitions can be characterized by the distance between the NBDs and are visually depicted in Figure 7. For Pgp to transition from an open-inward to an open-outward conformation and facilitate the successful translocation of a substrate to the extracellular (EC) region, it is necessary to overcome the distance between the NBDs. Our structurally-gated model aligns well with the efflux ratios previously reported for TPT, SN-38, and IRT [31,32]. Furthermore, this model harmonizes well with the biochemical data presented in this study. For instance, previous studies established that Pgp-mediated ATPase activation may depend on ligand-induced Pgp conformational changes [54,92]. The highest and lowest Pgp activation are likely when drugs induce open-outward and open-inward Pgp conformations, respectively. Thus, we theorize that the distance between the NBDs acts as a barrier that influences the transport of substrates. Substrates that reduce the conformational barrier, such as TPT, may be transported across the lipid bilayer at a faster rate, perhaps even without the binding of ATP. However, ATP binding and its subsequent hydrolysis would likely be required to provide the energy required to reset the transporter for the catalytic cycle to resume. Interestingly, all the tested CPTs appear to slightly reduce the Pgp-coupled ATP hydrolysis below basal levels. Studies have revealed that the relatively high basal ATPase activity for Pgp could be attributed to the presence of endogenous substrates, such as cholesterol, in the lipid bilayer [93,94]. These endogenous substrates are not transported by Pgp [95] but may be flip-flopped or shuttled between the two leaflets of the lipid bilayer by the protein [96,97]. Thus, the detected ATPase activity of Pgp comprises both the basal activity prompted by endogenous substrates and the ATP hydrolysis induced by exogenous ligands. Following this logic, if a ligand interacts with the same sites as these endogenous substrates or binds to and restricts the flip-flop, it will seem to impede ATPase activity if its rate of ligand-induced ATP hydrolysis is slower than that demonstrated by the endogenous substrates. This theory is supported by earlier studies that suggest that the rate of Pgp-mediated ATPase activity is a function of a complex interplay between ligands, membrane lipids, and the protein [40] and the fact that Pgp exhibits high basal ATPase activity [94]. We postulate that CPTs only appear to inhibit ATPase activity due to this phenomenon. Further research aimed at identifying the binding sites of both the ligands and endogenous substrates is needed to confirm this theory. The *K_m_* for drug-induced Pgp-mediated ATPase kinetics curve (Figure 2) revealed that TPT induced approximately 2- and 3-fold higher Pgp-ATP affinity than SN-38 and IRT, respectively. In terms of *V_max_*, the order of Pgp-mediated ATP hydrolysis among the tested drugs was TPT > SN-38 > IRT. The analysis of Pgp-ligand binding affinity, as determined by the quenching of the protein’s tryptophan residues, indicated that TPT exhibits the highest affinity for the protein, followed by SN-38. However, the differences in the binding affinity of CPTs to Pgp observed were minor and are unlikely to be the primary driving mechanism underlying the differential transport rates reported for these drugs.

In the presence of AMPPNP, the affinities of TPT, SN-38, and IRT for Pgp diminished by over threefold, which suggests that the binding of AMPPNP non-competitively inhibits the binding of CPTs and, perhaps, enhances the release of the drugs from the protein-drug complex state to the extracellular environment. Previously reported data suggest that the binding of ATP analogs are sufficient to switch Pgp from a high to a low drug affinity conformation [98,99]. Based on this analogy, it is likely that the binding of AMPPNP to Pgp either partially occludes or changes the local environment of the binding site of CPTs, thereby decreasing the protein-drug binding affinity. The changes in the drug binding sites may also be due to nucleotide-induced conformational changes in the entire Pgp structure [90] rather than localized at the binding site. These hypotheses are supported by both the absence of substrate and the significant rearrangement of residues in the central binding cavity of Pgp observed in the solved open-outward crystal structure of Pgp [70]. In the presence of saturating AMPPNP, both SN-38 and IRT induced significant changes in the protein conformation by shifting the protein into an open-outward state (Figure 4). On the other hand, there was no notable change in the TPT-induced Pgp conformation in the presence of AMPPNP. The lack of observable change in the presence of AMPPNP may be due to the protein having already shifted to a predominantly open-outward conformation in the presence of TPT. Thus, unlike SN-38 and IRT, which require the binding of ATP to shift Pgp into an open-outward conformation, TPT may be able to achieve this feat without ATP. This phenomenon may account for the differences in the transport rates observed for these drugs [31,32] However, further ligand-induced conformational studies are needed to confirm this theory. Remarkably, in the presence of saturating AMPPNP, all the tested CPTs induced a similar open-outward conformation in Pgp (Figure 4), highlighting the important role nucleotides play in the catalytic cycle of the protein.

The magnitudes for both the STDD amplitudes and the associated amplification factors estimated from the 1D H^1^ STDD NMR analysis were higher for TPT than IRT, implying that TPT has a relatively higher binding affinity to Pgp (Figure 5). These observations align with the results from the binding affinity (Figure 3), which indicate that TPT exhibits a slightly higher affinity for Pgp. Despite the E-ring of CPTs (depicted in Figure 1) being widely considered the functional group responsible for the binding of CPTs to the DNA-topoisomerase-1 complex [100], the groups responsible for drug-Pgp interactions remain elusive. The amplification factors derived from STDD NMR experiments are contingent upon NOE (Nuclear Overhauser Effect) transfer, a process that is inversely linked to distance. Among the various protons, those originating from C_15_ (as depicted in Figure 1 and Figure 5) exhibited the highest interactions with Pgp. Altering these functional groups alone or along with others that displayed noticeable interactions with the transporter could lay the foundation for developing the next generation of CPTs that can effectively evade Pgp-mediated efflux. While the prospect of developing enhanced drugs through this approach is promising, it comes with a limitation: the potential loss of drug functionality. Nonetheless, our understanding of the specific functional groups responsible for binding to Pgp, coupled with the existing structure-activity relationship studies involving CPTs and topoisomerase-DNA complexes, could lay the foundation for the development of potent next-generation CPTs capable of evading the transporter. To our knowledge, this study is the first to identify functional groups on TPT and IRT that likely interact with Pgp.

In summary, our results suggest that drug-induced Pgp conformational changes modulate Pgp-mediated transport of CPTs. Of the three drugs, TPT induces the highest conformational change in the protein by shifting Pgp from open-inward to open-outward. These TPT-induced conformational changes reduce the NBD conformational barrier (the distance between the NBDs) and, subsequently, stimulate transport. In comparison, SN-38 shifts the protein to an intermediate state between open-inward and open-outward, thereby moderately reducing the conformational barrier. Subsequently, SN-38 is modestly transported by Pgp. Unlike the other two drugs, IRT shifts the protein closer to the open-inward conformation with a relatively wider NBD distance and is poorly transported. Our study provides an underlying mechanism modulating Pgp-mediated transport of the CPTs while offering clues for rational drug design aimed at developing potent next-generation CPTs capable of evading Pgp.

## 4. Methods

### 4.1. Chemical Reagents

Irinotecan hydrochloride salt trihydrate and SN-38 were obtained from TCI (Montgomeryville, PA, USA). Topotecan was obtained from AstaTech (Bristol, PA, USA). Adenosine 5′-(β,γ-imido)triphosphate lithium salt (AMPPNP), acrylamide, ammonium chloride (NH_4_Cl), and 4-(2-hydroxyethyl)-1-piperazine ethane sulfonic acid (HEPES) were acquired from Sigma-Aldrich (Milwaukee, WI, USA). Nitrilotriacetic acid (NTA) and diethylaminoethyl cellulose (DEAE) resin were purchased from Thermo-Fisher Scientific (Waltham, MA, USA). Ethylene glycol tetraacetic acid (EGTA) and imidazole were obtained from Alfa Aesar (Tewksbury, MA, USA). Dithiothreitol (DTT) was purchased from Gold Biotechnology (Olivette, MO, USA). Sodium orthovanadate (Na_3_VO_4_) was purchased from Enzo Life Sciences (Farmingdale, Long Island, NY, USA). The detergent, n-dodecyl-β-D-maltoside (DDM), was acquired from EMD Millipore Corporation (San Diego, CA, USA). Disodium ATP, cholesterol, and Tris-HCl were purchased from Amresco (Solon, OH, USA). *Escherichia* (*E. coli*) total lipid extract powder was purchased from Avanti Polar Lipids Inc. (Alabaster, AL, USA). Dimethyl sulfoxide-d6 99.9 atom% D and Deuterium oxide 99.9 atom% D were obtained from Sigma Aldrich, Burlington, Ma. N-acetyl-L-tryptophanamide (NATA) was purchased from Sigma Aldrich (Milwaukee, WI, USA). All other reagents and chemicals not mentioned above but used in this study were purchased from Thermo-Fischer Scientific, unless otherwise stated.

### 4.2. Expression and Purification of P-Glycoprotein

Strains of *Pichia pastoris* genetically engineered to overexpress histidine-tagged wild-type mouse Pgp (MDR3) were cultured in glycerol media and further induced with methanol to enhance the overexpression of the protein [101,102] at the Bioexpression and Fermentation Facility (BFF), University of Georgia, Athens, GA, USA. The cells were collected and lysed using a repeated cycle of freezing in liquid nitrogen, blending, and thawing [103]. The protein was then purified using a two-step approach previously described with modification [101,104]. The first step involved an affinity chromatography technique with nickel-nitrilotriacetic acid (Ni-NTA) (ThermoFisher Scientific, Waltham, MA, USA), followed by ion-exchange chromatography with diethylaminoethyl cellulose (DEAE) resin (ThermoFisher Scientific). Next, the purified proteins were solubilized in DDM and concentrated using Amicon Ultra-15 100 kDa cut-off filters (EMD Millipore, Billerica, MA, USA). The yield and concentration of the purified protein were determined using an extinction coefficient of 1.28 per mg mL^−1^ at 280 nm on a Nanodrop (DeNovix, PHL) [104]. An SDS/PAGE analysis showed a purity of over 95% [101]. The DDM-solubilized proteins were stored at −80 °C.

### 4.3. Reconstitution of Pgp into Liposomes

Liposomes composed of 80% *w*/*v* Avanti *E. coli* total lipid extract and 20% *w*/*v* cholesterol, mixed in chloroform to a final concentration of 10 mg ml^−1^ and a volume of 10 mL, were made. The organic mixture was dried using a Buchi Rotavapor, Model R-114, and re-suspended with 10 mL of rehydration buffer (0.1 mM EGTA and 50 mM Tris-HCl solution with a pH of 7.4). The sample was freeze-thawed ten times using liquid nitrogen and a water bath to form liposomes of varying sizes, which were then extruded through a 400 nm cut-off filter at least 11 times using the LIPEX extruder (Northern Lipids, Burnaby, British Columbia, Canada) to produce homogeneously sized liposomes. Dynamic Light Scattering (DLS) was used to assess the sizes and uniformity of the extruded liposomes using a Malvern Zetasizer Nano ZS (Malvern Instruments Ltd., Malvern, UK). The DLS chromatograms were analyzed with Zetasizer 7.03 software and showed homogenously sized liposomes with an average size of 250 nm.

Approximately 50 µL of Pgp solubilized in detergent was dialyzed against HEPES buffer (20 mM HEPES, 100 mM NaCl, 5 mM MgCl_2_, 2 mM DTT, pH 7.4) for 2 h to remove residual detergents. The dialyzed Pgp was mixed with 4 mg mL^−1^ of the previously extruded liposomes and incubated on ice for 1 h to promote protein integration into the liposomes. Following incubation, the sample was dialyzed again in HEPES buffer for 2 h to remove excess detergents and further enhance protein integration into the liposomes to form proteo-liposomes. The concentration of the proteo-liposomes was determined using a Bio-Rad DC Protein assay, and aliquots were stored at −80 °C in HEPES buffer, pH 7.4.

### 4.4. Pgp-Mediated ATPase Activity Assay

Pgp-mediated ATP hydrolyses in the presence of TPT, IRT, and SN-38 were measured using Chifflet’s procedure as previously described [105] with some modifications. The Chifflet method estimates the activity of Pgp as a function of the concentration of inorganic phosphate, Pi, produced. Pi produces complexes with molybdenum, which characteristically has a high absorbance at 850 nm. The absorbance was measured with a FlexStation (III) spectrometer (Molecular Device, Sunnyvale, CA) on a 96-well plate. Approximately 250 nM of reconstituted Pgp in Chifflet buffer (150 mM NH_4_Cl, 5 mM MgSO_4_, 0.02% wt/vol NaN_3_, 50 mM Tris-HCl, pH 7.4) were incubated at 37 °C in the presence of varying concentrations of TPT, IRT, and SN-38 to determine the drug-induced ATPase activity of Pgp. The final concentrations of the reconstituted Pgp and ATP used were 50 nM and 3.2 mM, respectively. Control experiments containing vanadate (OVO_3_) and increasing concentrations of verapamil were run parallel to the samples. The ATPase activity kinetic curves were fit to a modified Michaelis-Menten equation (Equation (1)) [106], and Igor 6.2 pro software (Wavemetrics, Tigard, OK, USA) was used for analysis.

(1) $$V=\frac{V_{max}\left[S\right]}{K_{m}+\left[S\right]}+V_{basal}$$

where V is the ATP hydrolysis rate, *V_max_* is the maximum ATP hydrolysis at saturating drug concentrations, [S] is the substrate concentration, *K_m_* is the Michaelis-Menten constant, and *V_basal_* is the basal ATPase activity. 

### 4.5. Pgp-Drug Binding Affinity Determined by the Quenching of Protein Fluorescence

The physical properties of intrinsic tryptophan residues in proteins allow them to selectively absorb and emit around 295 nm and 330 nm wavelengths, respectively. The binding of ligands to the protein can cause changes in the local environment experienced by the tryptophan residue(s) and subsequently affect the fluorescence intensity of the protein [49]. Intrinsic protein fluorescence quenching was used to determine the binding affinity of substrates/ligands, such as drugs and nucleotides, to Pgp as previously carried out [56,58,107]. The Olis DM 45 spectrophotometer (Olis Corporation, Bogart, GA, USA) was used to measure ligand-induced intrinsic tryptophan quenching of protein fluorescence. A 10 nm band-pass filter was placed on the excitation and emission paths of the spectrophotometer to mitigate the effect of Rayleigh bands on the observed fluorescence emission spectra. Sample solutions containing Pgp reconstituted into liposomes were diluted in Chifflet buffer supplemented with 2 mM DTT to 1 µM. Intrinsic protein fluorescence emissions were measured at 330 nm following excitation at 295 nm. Ligand-induced quenching of intrinsic protein fluorescence was corrected (*F_corrected_*) for inherent factors such as background fluorescence, dilution, and inner filter effects using Equation (2) below [51,107]:

(2) $$F_{corrected}=\left(F-B\right)10_{}^{\frac{\left(\varepsilon_{ex}b_{ex}+\varepsilon_{em}b_{em}\right)\left[Q\right]}{2}}$$

where *F* is the measured intrinsic protein fluorescence at 330 nm, [Q] is the quenching ligand concentration, *ε_ex_* is the extinction coefficient for excitation, and *ε_em_* is the extinction coefficient for emission. The excitation (*b_ex_*) and emission (*b_em_*) path lengths were determined to be 0.4 mm and 10 mm, respectively. Finally, *B* represents the background, the fluorescence obtained from the sample containing the solvent and the drug only. For TPT, the extinction coefficients at 295 nm and 330 nm were 6.074 mM^−1^ cm^−1^ and 7.41 mM^−1^ cm^−1^, respectively. The extinction coefficients of IRT at 295 nm and 330 nm were 6.02 mM^−1^ cm^−1^ and 7.23 mM^−1^ cm^−1^, respectively. The estimated extinction coefficients of SN-38 were 3.86 mM^−1^ cm^−1^ and 5.95 mM^−1^ cm^−1^ at 295 nm and 330 nm, respectively. The extinction coefficient of AMPPNP at 295 nm was 0.03 mM^−1^ cm^−1^ and nearly transparent beyond 300 nm.

Ligand-induced intrinsic protein fluorescence quenching often occurs via several mechanisms, the most common being static and dynamic quenching [51]. Static quenching occurs when the ligand/quencher forms a non-fluorescent complex with the fluorophore before excitation [51]. In dynamic quenching, ligand-induced fluorescence quenching is mediated by random collisions between the ligand and the fluorophore [51]. Several experimental methods could be used to determine the predominant mechanisms of quenching present. However, the effect of two or more different temperatures (25 °C, 30 °C, and 37 °C) on the ligand-induced quenching of protein fluorescence was used in this study due to its effectiveness. The type of quenching exhibited was mainly static for the three drugs used in this study. The corrected fluorescence (*F_corrected_*) quenching curves were fit into a modified Stern–Volmer equation below (Equation (3)).

(3) $$F_{corrected}=\frac{F_{o\left(corrected\right)}}{1+K_{sv}\left[Q\right]}+F_{unquenched}$$

where *F_o_*_(*corrected*)_ is the fluorescence in the absence of a quenching ligand, *F_unquenched_* is an offset related to unquenched fluorescence, [*Q*] is the concentration of the ligand, and *K_sv_* is the Stern–Volmer constant.

### 4.6. Drug-Induced Pgp Conformational Changes Probed Indirectly by Acrylamide Quenching of Protein Fluorescence

The interaction between a ligand and protein can result in changes in the tertiary conformation of the protein and subsequently affect the availability of solvent-accessible intrinsic tryptophan residues within the protein to the bulk solvent [62,108]. Acrylamide is a small polar collisional quencher that can be used to measure changes in the availability of solvent-accessible tryptophan residues within a protein [108]. Since acrylamide cannot penetrate the hydrophobic interior of the protein or lipid bilayer, changes in the fluorescence of tryptophan residues within the protein can help predict changes in the tertiary structure of proteins [63,109]. Acrylamide quenching of protein fluorescence was used to probe drug-induced Pgp conformational changes indirectly. For this assay, a solution containing 1 µM of Pgp reconstituted into liposomes was titrated with acrylamide. The preparation was carried out in Chifflet buffer (pH 7.4) supplemented with 2 mM DTT. The assay was performed both in the presence and absence of ligand(s). Pgp fluorescence was monitored at 330 nm following excitation at 295 nm as the sample solution was titrated with an increasing acrylamide concentration. The fluorescence intensities were corrected for dilution, background, and inner filter effects using Equation (3). A Stern–Volmer plot of *F_o_*_(*corrected*)_/*F*_(*corrected*)_ versus the concentration of acrylamide added was generated. The slope of the curve, an indicator of the degree of quenching, is related to the Stern–Volmer’s constant (*K_sv_*) by Equation (4) [51].

(4) $$\frac{F_{o\left(corrected\right)}}{F_{\left(corrected\right)}}=1+K_{sv}\left[Q\right]$$

where [*Q*] is the concentration of the quencher, acrylamide.

### 4.7. Atomic Force Microscopy Imaging

Atomic force microscopy (AFM) has been adapted for analyzing protein dynamics and structure, which has enabled the determination of conformational changes induced by ligands [79,110]. In this study, Pgp reconstituted into liposomes was diluted in imaging buffer (20 mM HEPES, 100 mM NaCl, and 5 mM MgCl_2_; pH 7.4) to 100 nM. About 90 µL droplets of the diluted Pgp solution were deposited on cleaned glass coverslips (Corning, Corning, NY, USA), followed by 30 min of incubation at 25 °C. This step caused the proteoliposomes to rupture, forming a planar lipid bilayer on top of the mica surface. The samples were rinsed at least five times using the buffer exchange technique, wherein 90 µL of buffer was utilized. Next, AFM imaging of the EC-side and C-side domains of Pgp protrusions above the lipid bilayer was performed as previously described [79]. For each of the selected CPTs used in this study, AFM imaging of the ligand-free Pgp (apoPgp) was performed first, then ligand was added, and imaging was repeated in nominally the same area. The amounts of TPT, SN-38, and IRT added were 60 µM, 30 µM, or 100 µM, respectively. Only membrane-external protrusions that could be positively assigned as either EC or C-side domain features of Pgp were analyzed. All the AFM images were obtained in an imaging buffer at about 30 °C in tapping mode with biolever mini tips (BL-AC40TS, Olympus, Tokyo, Japan) installed on a commercial instrument (Asylum Research Cypher, Santa Barbara, CA, USA). The tip-sample force was kept below 100 pN to minimize the probability of protein distortion. The AFM images were analyzed with custom software written in Igor Pro 7 (Wavemetrics, Portland, OR, USA), as described previously [79,111]. Kernel density estimation with Epanechnikov kernels in Igor Pro 7 software was used to generate smoothed histograms for further analysis [112]. The histograms were fitted with a summation of Gaussian distributions. The Bayesian information criterion was used to select the best model after fitting with 1–5 Gaussian distributions as described previously [113] (see Supporting Information).

### 4.8. Saturation Transfer Double Difference (STDD) NMR

STDD NMR is a robust ligand-focused technique used to empirically determine ligand-receptor interactions and epitope mappings [114,115]. STD NMR involves the application of a long, low-powered pulse to selectively saturate the protons of the receptor protein [115]. For a molecule that binds to the receptor, only the protons within a distance of 5 Å from the saturated protein receive noticeable saturation transfer [73]. The saturation transferred from the receptor to the bound ligands can be detected upon dissociation as a saturation transfer difference (STD). The observed degree of saturation transferred inversely correlates with the distance between the ligand and the receptor and reflects the degree of interaction. Due to the high lipophilicity of most Pgp substrates, there is a high possibility of non-specific interactions between the ligands and liposomes used, which can interfere with the STD NMR spectrum observed. The STD NMR spectrum of the drug in the presence of liposomes was performed and subtracted from the STD ^1^H NMR spectrum of the ligand in the presence of proteins reconstituted into liposomes to yield a STDD. This double subtraction accounts for the potential STD contributions from ligand-liposome interactions [116,117]. This technique was used to probe the functional groups of TPT and IRT involved in Pgp-drug interactions. All NMR experiments were carried out at 25 °C (unless otherwise stated) on a Varian INOVA 600 MHz NMR spectrometer installed with a 1H-detect broadband probe {HX}. The NMR data were analyzed with iNMR software (Nucleomatic, Molfetta, Italy), Igor Pro 6.2, and Mnova 14.2.0 (Mastrelab Research S.L., Santiago de Compostela, Spain). 

The STDD NMR procedure in this study was performed as described by [56,118] with some modifications. The samples for STDD NMR experiments were prepared with 100 mM potassium phosphate buffer [80% D_2_O, 20% ddH2O, pH 7.4] containing 1 µM Pgp reconstituted into liposomes and 800 µM of either TPT or IRT. The control samples were identically prepared with liposomes instead of Pgp reconstituted in liposomes. A tailored gradient pulse sequence, water suppression by gradient-tailored excitation (WATERGATE), was included to suppress the background water signals [119,120]. The protein was selectively excited and saturated with a train of 50 ms Gaussian-shaped-selective pulses for 2 s, followed by a relaxation delay of 5 s [71,114]. The STD NMR spectra were obtained via phase cycling by alternating the Pgp irradiation resonance between −1.5 ppm (on resonance) and 42 ppm (off-resonance) for a total of 512 scans [56]. The difference between the on-resonance and the off-resonance created the STD ^1^H NMR spectrum. A control experiment consisting of liposomes and drugs was obtained under identical conditions and subtracted from the ^1^H STD NMR spectrum of the reconstituted Pgp sample with the drug to generate the STDD ^1^H NMR spectrum. The difference observed (Δl) directly correlates with the degree of interaction between the functional groups of drugs and the protein. The STDD amplification factor was calculated using Equation (5) below [60,71].

(5) $$STDD\;Amplication\;Factor=\frac{\left[l\right]}{\left[P\right]}\frac{\Delta l}{I_{o}}$$

where *I_o_* is the amplitude of the ^1^H NMR peaks, $\left[l\right]$ is the ligand concentration, and $\left[P\right]$ is the protein concentration.

## Data Availability

Data are available upon request.

## Supplementary Materials

The following supporting information can be downloaded at: https://www.mdpi.com/article/10.3390/ijms242216058/s1.
