# Supplementary material for: Drug-Induced Conformational Dynamics of P-Glycoprotein Underlies the Transport of Camptothecin Analogs
Source: Int J Mol Sci. 2023 Nov 7;24(22):16058. doi: 10.3390/ijms242216058 (PMC10671697; doi:10.3390/ijms242216058)
Supplement: Supplementary file 1 [file ijms-24-16058-s001.zip › ijms-2673362-supplementary.pdf]

Supporting Information

Drug-induced Conformational Dynamics of P-Glycoprotein Underlies the Transport of Camptothecin Analogs

Authors

Gershon A. K. Mensah[1], Katherine G. Schaefer[2], Michael G. Bartlett[1], Arthur G. Roberts[1], Gavin M. King[2]

[1]Department of Pharmaceutical and Biomedical Science, University of Georgia, Athens, GA 30602

[2]Department of Physics and Astronomy, University of Missouri, Columbia, MO 65211

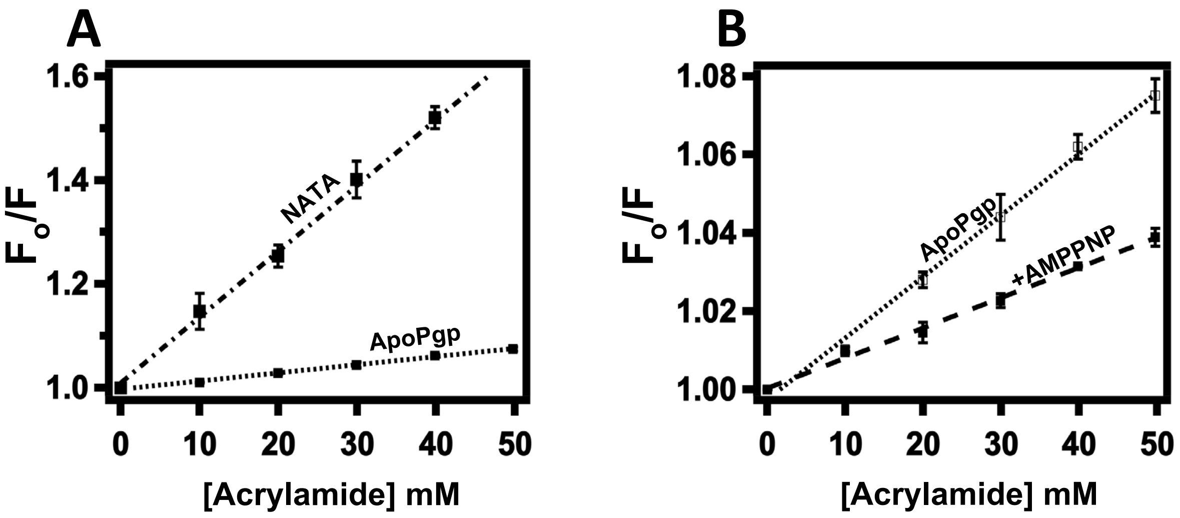

Supplemental Figure S1. Pgp conformational changes probed by acrylamide quenching of tryptophan residue fluorescence. (S1A) Stern-Volmer plot of NATA (dash-dotted line), and ApoPgp (dashes). (S1B) Stern-Volmer Plot of ApoPgp (dashes) and Pgp in the presence of saturating (3.2 mM) AMPPNP (short-dashes).

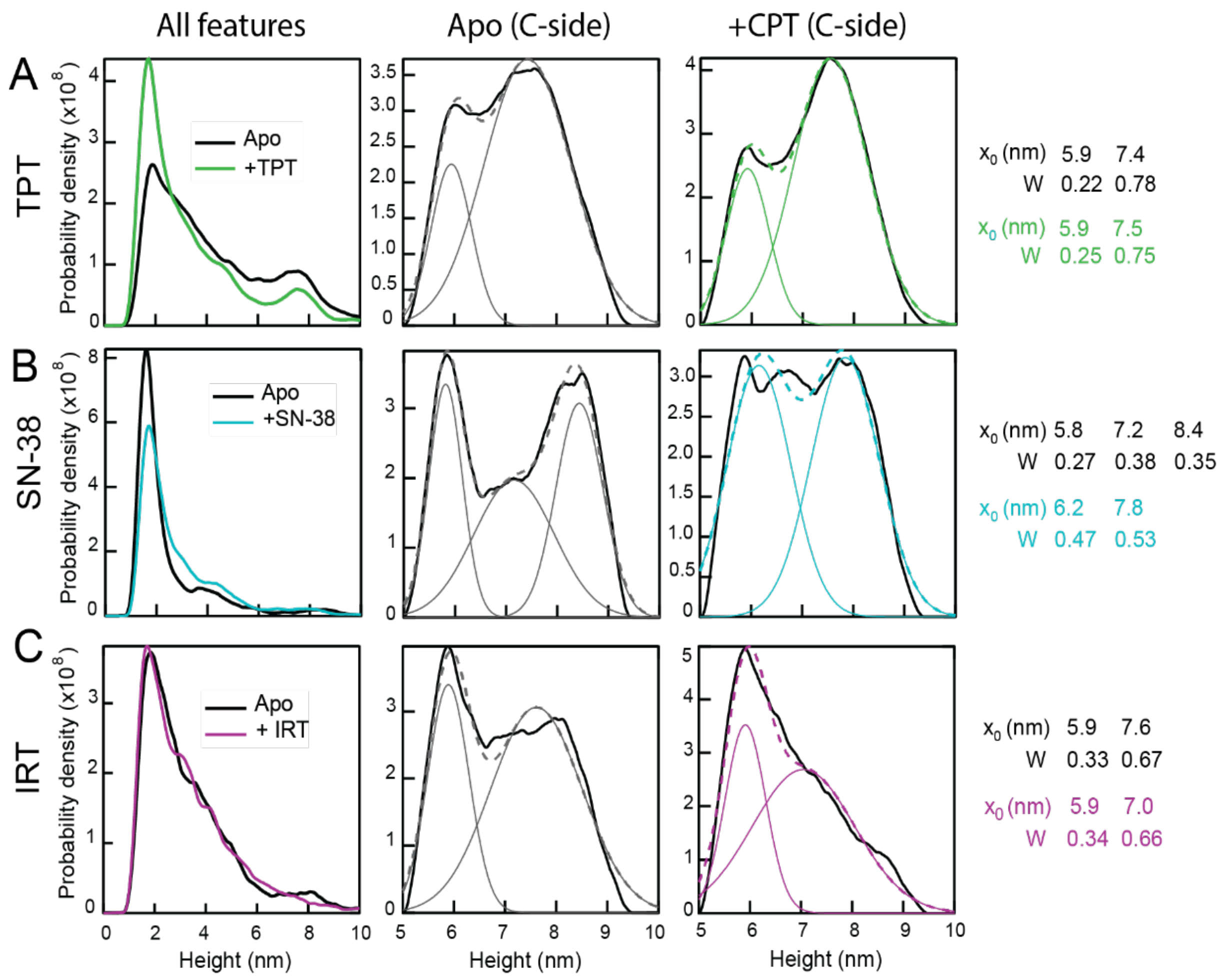

Supplemental Figure S2. Histograms of Pgp + CPTs. (A) TPT, (B) IRT, and (C) SN-38. The right panels show histograms of all analyzed particles. The C-side apo data (middle panels; solid black line) is fitted using the Bayesian information criterion to determine the appropriate number of Gaussian distributions. Individual Gaussian distributions (solid gray lines) are summed together to form the model fit (dashed gray lines). Left panels display the +CPT conditions. Raw data is portrayed as a black line, with individual Gaussians (solid-colored lines) and summary fits (dashed colored lines) overlaid. All peak positions and weights are presented to the left of the graphs.
